# Optimized ridge-furrow ratio regulated soil nutrients, promoted grain filling and increased wheat yield

**DOI:** 10.3389/fpls.2026.1776299

**Published:** 2026-03-04

**Authors:** Kun Liu, Yu Shi, Zhenwen Yu, Yongli Zhang, Zhen Zhang

**Affiliations:** State Key Laboratory of Wheat Improvement, Agricultural College, Shandong Agricultural University / Key Laboratory of Crop Physiology, Ecology and Cultivation, Ministry of Agriculture and Rural Affairs, Tai’an, China

**Keywords:** grain filling, grain yield, ridge-furrow ratio, soil nutrient, wheat

## Abstract

**Introduction:**

The ridge-furrow planting system improves wheat grain filling and soil quality; however, the effects of appropriate ridge-furrow ratio on soil nutrient dynamics and nutrient-plant interaction and its potential response mechanism under ridge-furrow planting mode are still unclear. This study was designed to determine the optimal ratio and clarify the coupling relationships among soil nutrient availability, grain filling, and grain yield.

**Methods:**

Through a two-year consecutive field experiment, the effects of traditional planting pattern (M1) and three ridge-furrow planting patterns (ridge-furrow ratio of 50 cm∶50 cm, 75 cm∶50 cm, and 100 cm∶50 cm; M2−M4) on grain filling, soil nutrient content, and grain yield of wheat were analyzed.

**Results:**

Ridge-furrow patterns with varying ratios significantly affected wheat soil nutrient uptake and grain yield. Compared with other treatments, M3 treatment increased the activities of soluble amylase and bound starch synthase in the middle and late filling. At 21–28 days post-anthesis across two growing seasons, M3 increased amylose, amylopectin, and total starch accumulation by 7.21–23.37%, 7.86–22.71%, 7.72–22.88% and 7.15–23.06%, 7.80–21.93%, 7.66–22.07%, respectively. M3 treatment increased the maximum and average grain filling rate during filling, promoted grain filling of wheat, and obtained the highest grain weight. Moreover, M3 treatment is beneficial to improve soil fertility and promote the accumulation of microorganisms, thus creating a favorable environment for plant growth. Finally, the grain yield of M3 was 3.12−8.68% and 4.79−10.91% higher than other treatments, respectively, achieving the highest grain yield.

**Discussion:**

In conclusion, our findings confirm that adopting the M3 ridge-furrow ratio is the optimal practice for winter wheat ridge-furrow cultivation in the North China Plain.

## 1 Introduction

As an important part of global food demand, ensuring the increase and stability of wheat production is crucial for global food security in the future (Fan et al., 2025). As the core winter wheat cultivation region in China, the North China Plain (NCP) makes up over 50% of China’s total winter wheat sown area, and the yield accounts for more than 60% of the total national winter wheat yield (Fu et al., 2025; Hu et al., 2025). Over the past few decades, augmented inputs of irrigation water and fertilizers have served as the dominant strategy for enhancing wheat productivity. However, traditional planting patterns usually lead to nutrient imbalance, thereby reducing food crop production (Shubha et al., 2025). Therefore, creating efficient planting methods to achieve efficient resource recycling and maintain soil fertility is crucial to achieving the goal of sustainable agriculture.

In agriculture, soil nutrients are essential for promoting crop growth, promoting the development of nutritional and reproductive structures, and ultimately increasing biomass production and yield. Different planting patterns have different effects on farmland, resulting in different soil environments and different effects on the storage and release of soil nutrients (Su et al., 2025). Ridge-furrow planting pattern increases soil water holding capacity, promotes root proliferation, and enhances the efficiency of water and nutrient uptake by adjusting farmland micro-topography (Xing et al., 2025). Zhang et al. (2025) demonstrated that ridge-furrow planting notably enhanced water utilization efficiency, nitrogen use efficiency, and grain yield of crops compared with the flat planting pattern. A large number of studies have confirmed that furrow planting improves soil quality by improving the hydrothermal environment and ultimately increases yield (Quan et al., 2024; Indoshi et al., 2025). Regarding the planting patterns of maize (Indoshi et al., 2022), potato (Sun et al., 2023), rapeseed (Wang et al., 2025d), the yield increase under ridge-furrow planting pattern was also verified. However, the improvement effect of ridge-furrow planting pattern on soil hydrothermal environment will have different responses to different ridge-furrow ratios (Zhang et al., 2021). Luo et al. (2021) reported that ridge-furrow ratios of 20 cm∶20 cm and 30 cm∶20 cm yielded the highest wheat productivity and precipitation use efficiency under rain-fed production conditions in East Africa. Qiang et al. (2022) concluded that when the ridge-furrow ratio was 40∶40 cm in the semi-arid region of the Loess Plateau, the accumulation of dry matter, crop yield and water productivity of wheat were the highest. Therefore, rational adjustment and optimization of the ridge-furrow ratio are essential for augmenting soil nutrient content, which in turn plays a pivotal role in improving wheat yield performance.

Previous studies have demonstrated that ridge-furrow planting patterns exert a primary influence on crop yield and soil hydrothermal conditions (Zhang et al., 2023; Liang et al., 2024). However, there are limited studies on the coupling mechanism between soil nutrients and grain filling dynamics in NCP. Therefore, a two-year wheat planting experiment was carried out in the NCP, aiming to (1) explore the impacts of varying ridge-furrow ratios on soil nutrient changes, grain filling and yield of wheat; (2) Analyze the potential yield-increasing pathways of optimizing the ridge-furrow ratio under the ridge-furrow planting system, and quantify the key factors governing crop yield formation; (3) The coupling mechanism mediating the interactions between rhizosphere microorganisms and the physiological regulation of aboveground grain filling was discussed. The results of this study provide a robust reference framework for the construction of high-yield, high-efficiency, and sustainable wheat cultivation regimes in the NCP.

## 2 Materials and methods

### 2.1 Experimental site description

**Figure 1:**
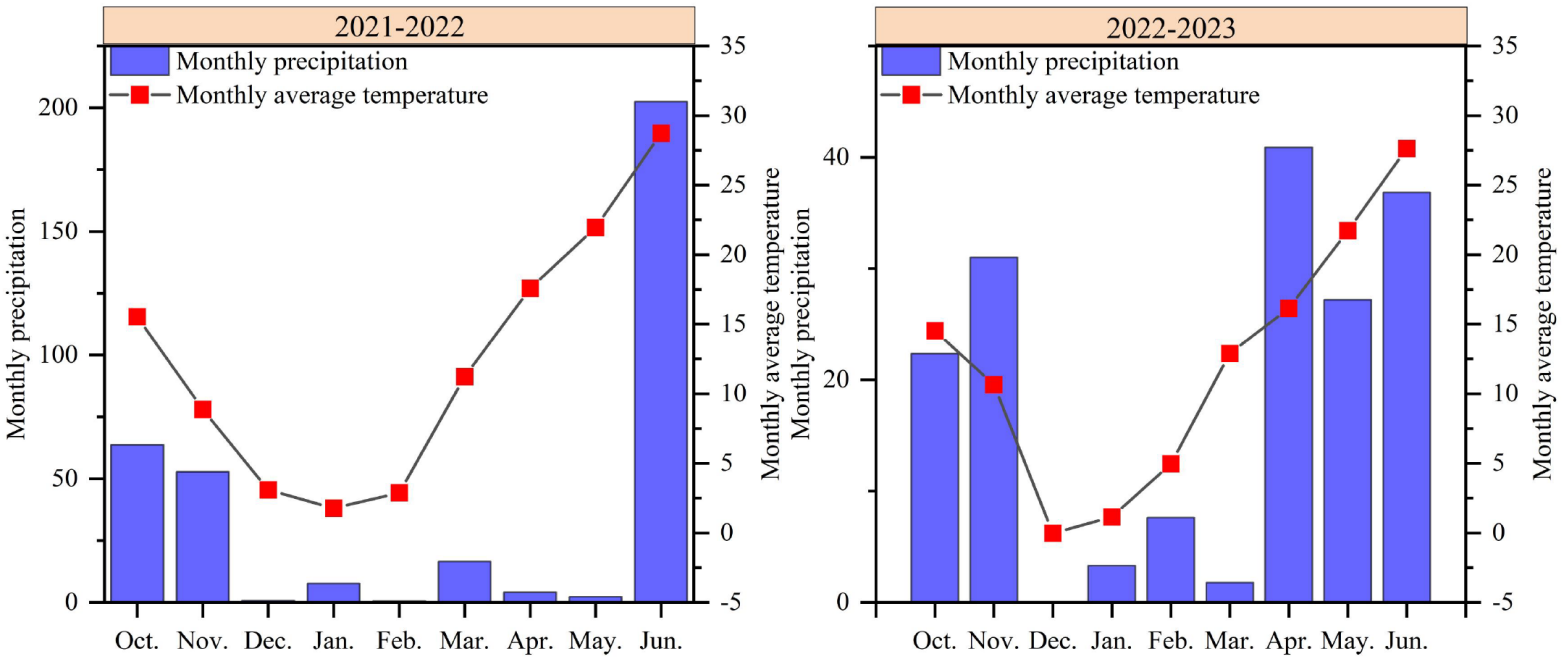
Monthly precipitation and monthly mean temperature during the experimental period.

Field experiment was conducted at Xiaomeng Experimental Station (35° 40 ′ N, 116° 24 ′ E) in Jining, China, during the wheat season from 2021 to 2023. The study site is located in a temperate monsoon climate zone, with loam as the dominant soil type; all field trials were conducted on the same experimental plot. Prior to the experiment, the properties of the topsoil (0−20 cm depth) were determined as follows: soil organic matter (SOM) 14.79 g·kg^-1^, total nitrogen (TN) 1.23 g·kg^-1^, alkali-hydrolyzable nitrogen (AN) 120.30 mg·kg^-1^, available phosphorus (AP) 30.86 mg·kg^-1^, and available potassium (AK) 119.60 mg·kg^-1^. Monthly precipitation and monthly mean temperature throughout the experimental period are presented in **Figure 1**.

### 2.2 Experimental design and treatments

**Figure 2:**
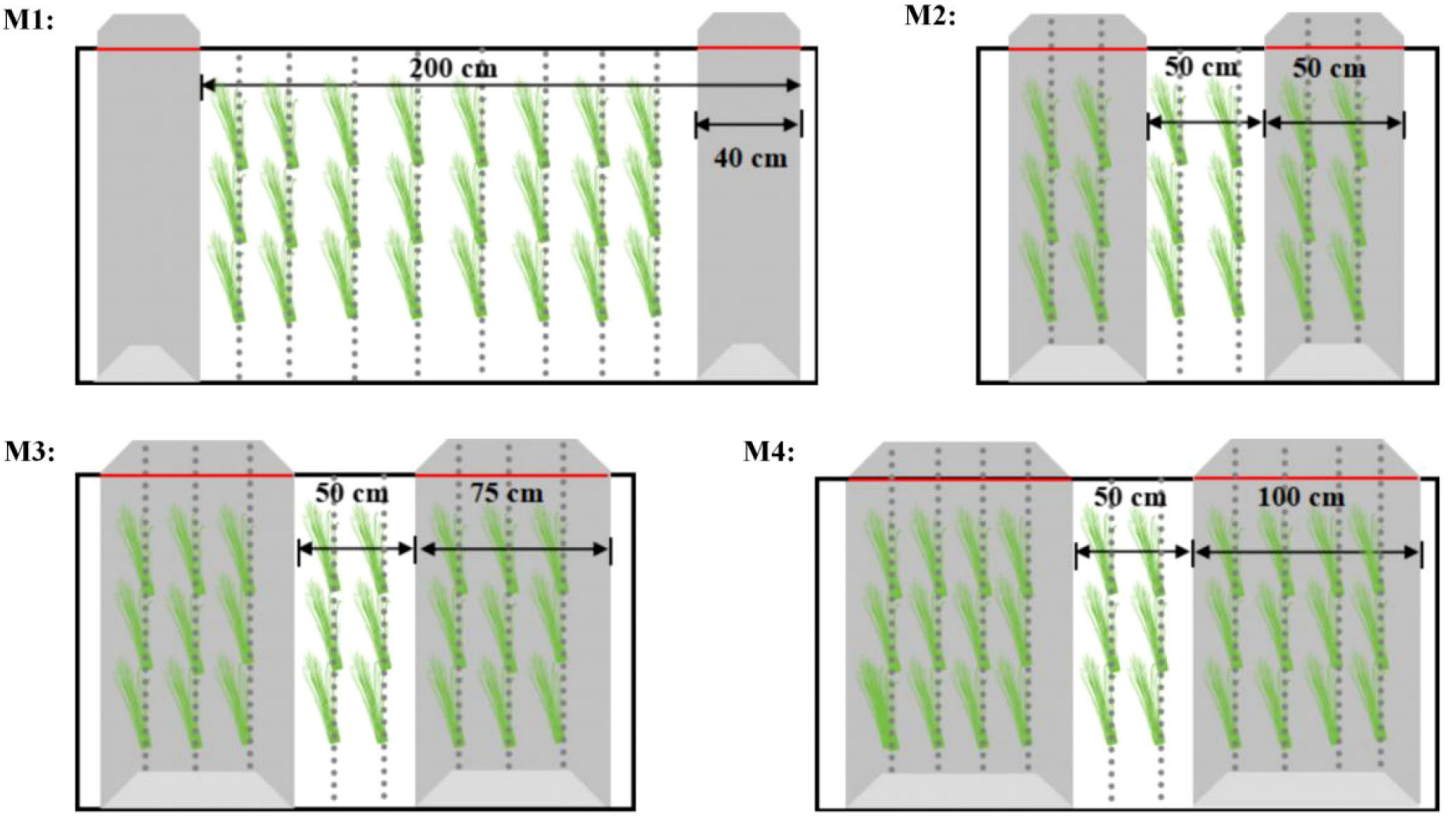
Planting pattern.

The experiment followed a randomized block design, with each treatment replicated three times. The treatments included the traditional planting pattern (M1) and ridge-furrow planting pattern with ridge-furrow ratio of 50 cm∶50 cm (M2), 75 cm∶50 cm (M3) and 100 cm∶50 cm (M4), as illustrated in **Figure 2**. Each plot had an area of 30 m×4.5 m. The project utilized ‘Jimai 22’ as a wheat variety. Wheat was sown at a density of 330 plants m^-2^ on October 24, 2021, and 270 plants m^-2^ on October 18, 2022, with sowing conducted on both ridges and furrows. Harvesting was carried out on June 11 of the following year for both growing seasons. Phosphorus fertilizer (P_2_O_5_) and potassium fertilizer (K_2_O) of 150 kg ha^-1^ and nitrogen fertilizer of 105 kg·ha^-1^ were applied before sowing, and used a rotary tiller to incorporate it into the soil. Nitrogen fertilizer of 135 kg ha^-1^ was applied between wheat rows at jointing. Furrow irrigation was adopted, and the specific irrigation amounts are detailed in **Table 1**. Other field management measures, including pesticide and herbicide application, were implemented in accordance with local conventional operational standards.

**Table 1:** Irrigation amount during the experiment.

Year	Treatment	Jointing	Anthesis	Total irrigation
		(mm)	(mm)	(mm)
2021–2022	M1	75.40	62.83	138.23
	M2	64.60	53.10	117.70
	M3	57.60	45.34	102.94
	M4	50.51	39.78	90.29
2022–2023	M1	44.23	37.78	82.02
	M2	32.23	32.67	64.90
	M3	28.68	29.07	57.75
	M4	26.57	27.00	53.57

### 2.3 Measurements

#### 2.3.1 Soil nutrient content and microbial biomass nitrogen content

Samples were taken from the 0–40 cm soil depth by soil drilling at wheat maturity, 20 cm per depth, and repeated three times. Soil samples were sampled and mixed from all rows on the ridge, all rows in the furrow and all rows in the traditional planting pattern, respectively, and repeated three times. All samples were stored in a refrigerator at −80 °C for later use. According to the method of Truong and Marschner (2018), the content of soil microbial biomass nitrogen (MBN) in mature period was determined by chloroform fumigation extraction method. Referring to the method of Wang et al. (2025b), the soil nutrient content at maturity was determined. Soil TN, SOM, AN, AP, and AK were determined by Kjeldahl nitrogen digestion method, potassium dichromate-concentrated sulfuric acid external heating method, diffusion method, NaHCO_3_ extraction-Mo-Sb colorimetric spectrophotometric method and the NH_4_OAc extraction-flame photometric method, respectively.

#### 2.3.2 Grain filling characteristics

Wheat spikes were tagged on the day of anthesis, and 20 tagged spikes were sampled every 7 days thereafter. Initially, the samples were subjected to heat inactivation at 105 °C for 10 min; thereafter, oven drying at 70 °C was implemented until a stable weight was attained, which preceded the weighing operation. The Logistic growth equation was employed to characterize the grain-filling process. The equation is: y = a/(1+be^-cx^), where x is the number of days after anthesis, y is the grain weight, and a, b, c are coefficients of the Logistic growth equation. For this analysis, four secondary parameters were used to describe the grain-filling characteristics: maximum filling rate (Vmax), time to reach maximum filling rate (Tmax), average accumulation rate (Vmean), and grain-filling duration (T).

#### 2.3.3 Starch accumulation and amylase activity in grains

Wheat spikes were tagged on the day of anthesis, and 40 tagged spikes were sampled every 7 days thereafter. The 20 collected samples was quick-frozen in liquid nitrogen and preserved at −80 °C for subsequent analysis. Crude enzyme extracts were prepared using the method reported by Wang et al. (2021), while the activities of soluble starch synthase (SSS) and granule-bound starch synthase (GBSS) were assayed according to the protocol established by Yang et al. (2003). The absorbance value was recorded at the wavelength of 340 nm, and the key enzyme activity of starch synthesis was determined by adding 0.01 OD value per minute to 1 enzyme activity unit (U). Another subset of the spike samples was subjected to heat inactivation at 105 °C for 10 minutes, then dried at 70 °C to constant weight, and then weighed. The assay for amylose and amylopectin contents in wheat grains was performed based on the standardized procedure developed by Wang et al. (2021). Starch accumulation was calculated as the product of starch content and grain weight. Total starch accumulation (AS) in wheat grains was defined as the sum of amylose accumulation (AAM) and amylopectin accumulation (AAP).

#### 2.3.4 Grain yield

The methodology employed in this study was based on the work of Si et al. (2023), harvesting operations were carried out on every 4-m wheat row within each experimental plot when the crop reached physiological maturity. Air-drying was performed on the harvested grains to achieve a consistent moisture level of 13%, then individually weighed to determine the mean value, from which the final grain yield was calculated.

### 2.4 Statistical analyses

IBM SPSS Statistics 26.0 (IBM Corp, Armonk, NY, USA) was employed to carry out all statistical analyses. ANOVA was executed separately for each growing year with a degree of freedom (df) of 3. Multiple comparisons were implemented via the LSD test with a significance threshold of α=0.05, and differences between treatments were denoted using the letter grouping method. Pearson correlation analysis was carried out to determine P-values. Additionally, a linear regression model was employed to fit the relationships between MBN and TN, SOM, AN, AP, and AK. All data were visualized using Origin 2024 (OriginLab Corporation, Northampton, MA, USA). Values associated with the M2, M3, and M4 treatment groups were derived by means of the weighted average methodology (Nader, 2016). Ridges and furrows are represented by -R and -F, respectively.

## 3 Results

### 3.1 Soil nutrient content in 0−40 cm soil depth

**Figure 3:**
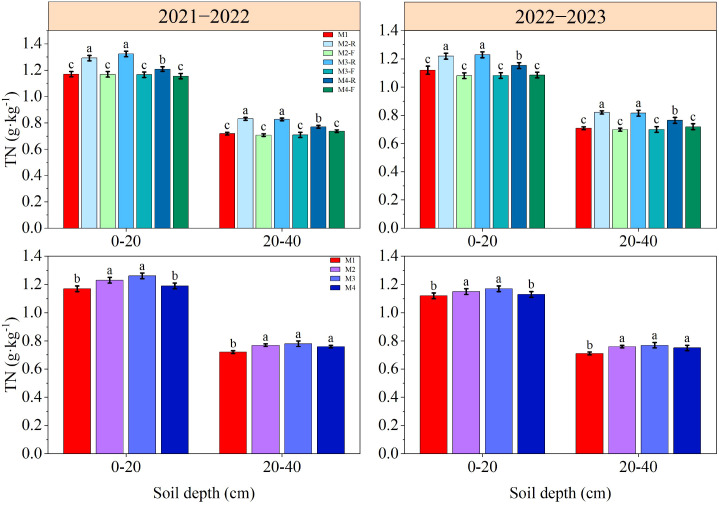
Effects of different treatments on soil total nitrogen at wheat maturity in 2021−2022 and 2022−2023. -R and -F denote the ridge and furrow of the treatment, respectively. Distinct letters following the data values represent statistically significant differences across treatments at *P* < 0.05 (n = 3). Vertical bars denote the mean ± standard error.

**Figure 4:**
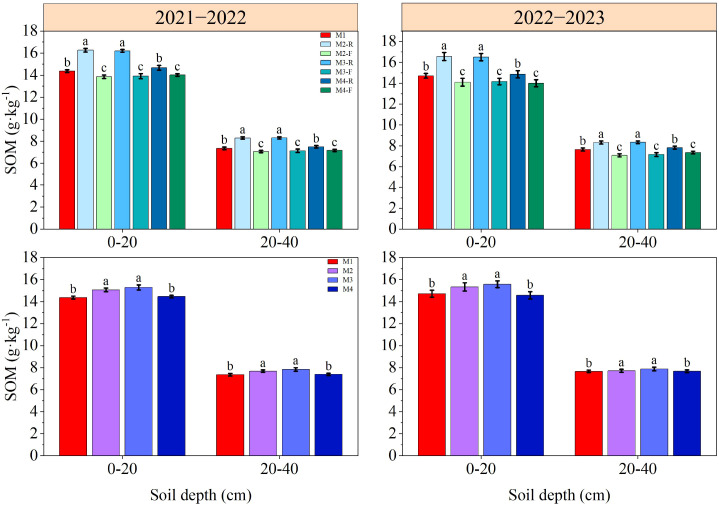
Effects of different treatments on soil organic matter at wheat maturity in 2021−2022 and 2022−2023. -R and -F denote the ridge and furrow of the treatment, respectively. Distinct letters following the data values represent statistically significant differences across treatments at *P* < 0.05 (n = 3). Vertical bars denote the mean ± standard error.

**Figure 5:**
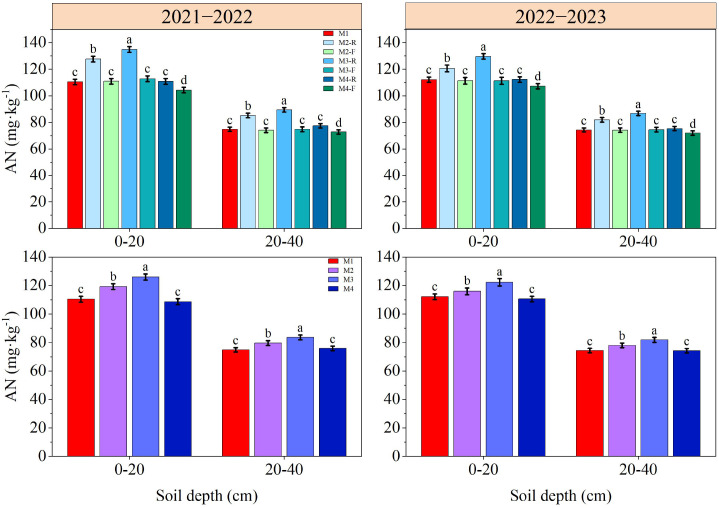
Effects of different treatments on soil alkali-hydrolyzed nitrogen at wheat maturity in 2021−2022 and 2022−2023. -R and -F denote the ridge and furrow of the treatment, respectively. Distinct letters following the data values represent statistically significant differences across treatments at *P* < 0.05 (n = 3). Vertical bars denote the mean ± standard error.

**Figure 6:**
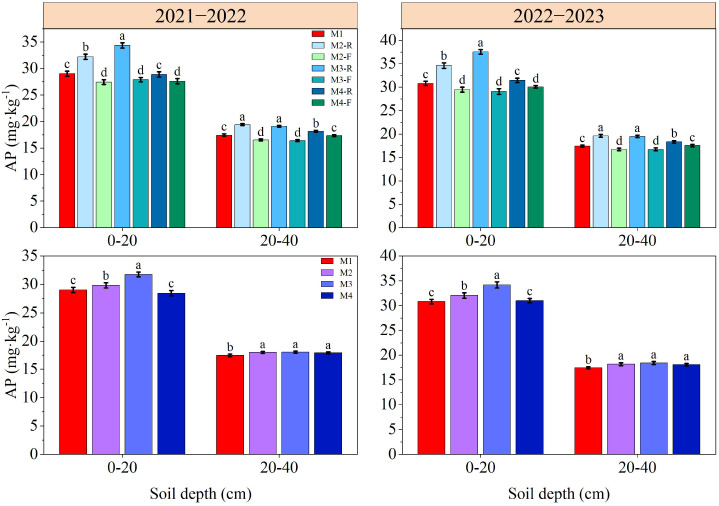
Effects of different treatments on soil available phosphorus at wheat maturity in 2021−2022 and 2022−2023. -R and -F denote the ridge and furrow of the treatment, respectively. Distinct letters following the data values represent statistically significant differences across treatments at *P* < 0.05 (n = 3). Vertical bars denote the mean ± standard error.

**Figure 7:**
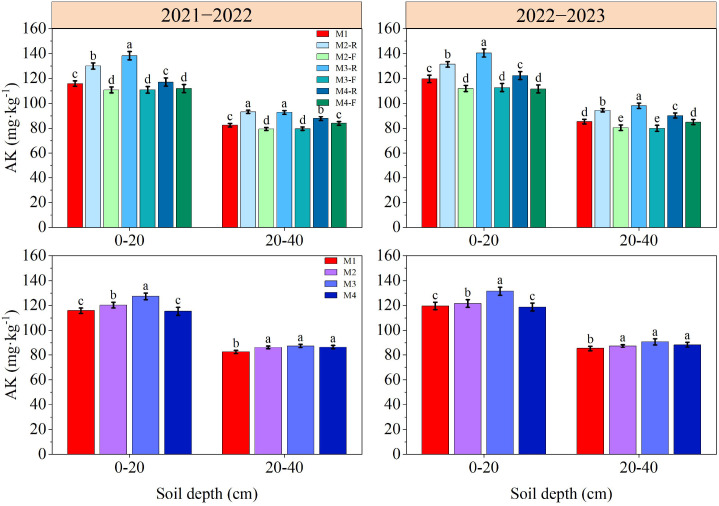
Effects of different treatments on soil available potassium at wheat maturity in 2021−2022 and 2022−2023. -R and -F denote the ridge and furrow of the treatment, respectively. Distinct letters following the data values represent statistically significant differences across treatments at *P* < 0.05 (n = 3). Vertical bars denote the mean ± standard error.

The contents of SOM, TN, AN, AP and AK in soil decreased gradually with the deepening of soil depth (**Figures 3**–**7**). In the 0−40 cm soil depths, the contents of TN, SOM, AN, AP, and AK under the M3-R treatment were the highest among all treatments; the values in question were markedly higher compared with those observed in other treatments (*P* < 0.05), with the exception of TN and SOM contents under the M2-R treatment. Across the two growing seasons, the TN and SOM contents in the 0−40 cm soil depths under the M2 and M3 treatments were significantly higher than those under the M1 treatment (*P* < 0.05). In the 2021−2022, relative to other treatments, the AN content under the M3 treatment was elevated by 5.68−16.00% (0−20 cm soil depth) and 5.01−11.66% (20−40 cm soil depth), respectively. In the 2022−2023, the corresponding increments in AN content were 5.46−10.59% and 5.04−10.25%, respectively. With respect to AP content, the M3 treatment induced an increase of 6.44−13.27% in the 0−20 cm soil depth relative to other treatments in the 2021−2022, and this increment expanded to 12.82−36.39% in the same soil depth during the 2022−2023. During the 2021–2022 cultivation season, for the 0–20 cm soil depth, the AK content of the M3 treatment exceeded those of the M1, M2, and M4 treatments by 9.91%, 5.79%, and 10.38%, respectively. In the 2022−2023, the respective increases in AK content in the same soil depth were 11.79%, 5.51%, and 10.76% relative to the M1, M2, and M4 treatments. Collectively, these results indicate that the M3 treatment can effectively improve soil nutrient status.

### 3.2 Soil microbial biomass nitrogen content

**Figure 8:**
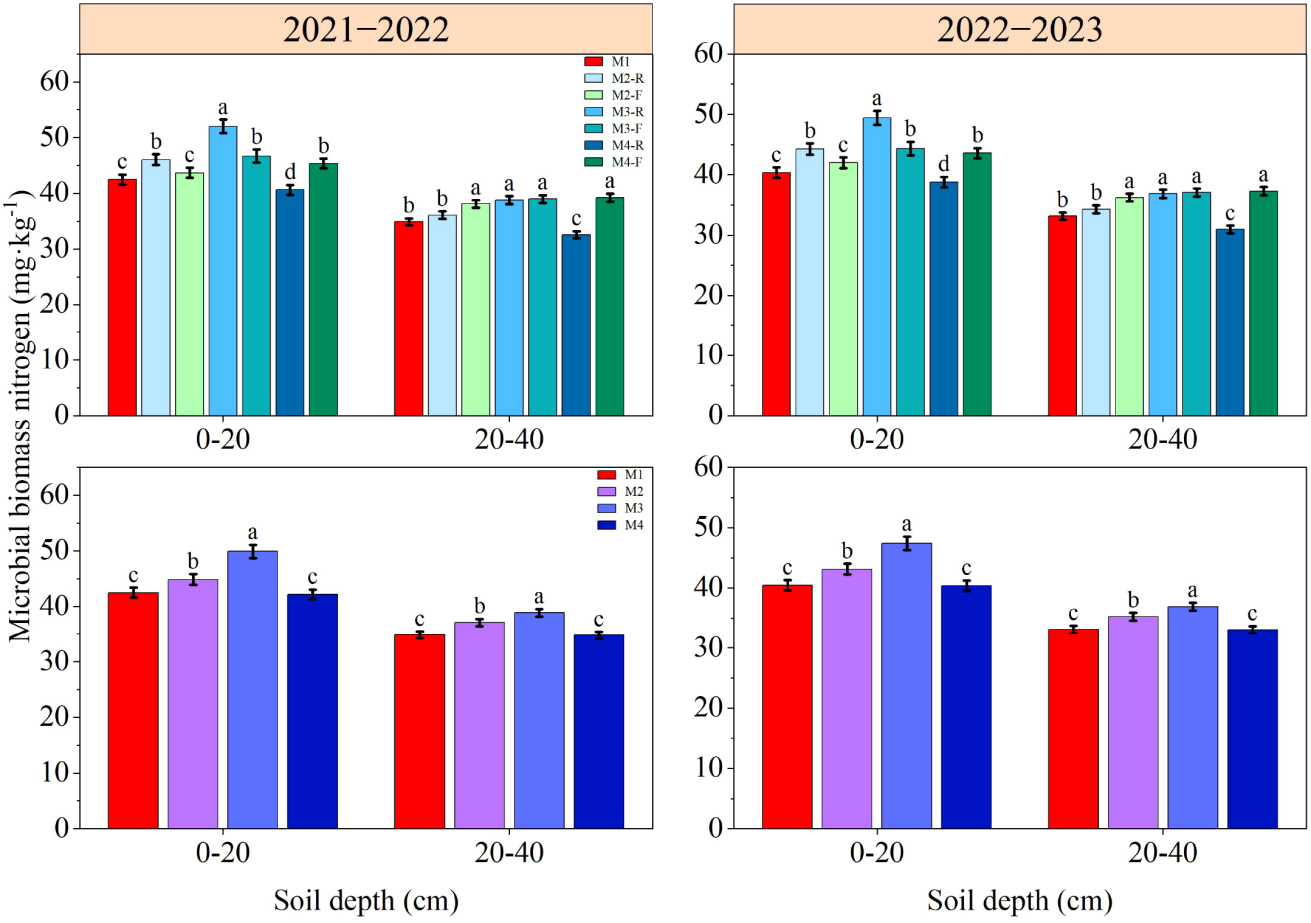
Effects of different treatments on soil microbial biomass nitrogen at wheat maturity in 2021−2022 and 2022−2023. -R and -F denote the ridge and furrow of the treatment, respectively. Distinct letters following the data values represent statistically significant differences across treatments at *P* < 0.05 (n = 3). Vertical bars denote the mean ± standard error.

Ridge-furrow planting patterns with varying ridge-furrow ratios exerted a significant influence on soil MBN content (**Figure 8**). Across both growing seasons, the MBN concentration in the topsoil (0–20 cm depth) subjected to the M3-R treatment exceeded that of every alternative treatment by a significant margin (*P* < 0.05). For the 20−40 cm soil depth, MBN levels in the M3-R treatment was exceeded those in the M1, M2-R, and M4-R treatments significantly over the two growing seasons; by contrast, no statistically significant differences were detected between the M3-R and the M2-F, M3-F, M4-F treatments. In the 2021−2022 growing season, the MBN content in the 0−20 cm soil depth under the M3 treatment was 11.19−18.23% higher than that under other treatments. This increment ranged from 9.90% to 17.36% in the 2022−2023 growing season. Moreover, during both growing seasons, MBN levels in the 20−40 cm soil depth under the M3 treatment was significantly greater than those observed in every alternative treatment (*P* < 0.05). These findings show that the M3 treatment is conducive to enhancing soil microbe activity and hastening the breakdown of soil organic matter.

### 3.3 Relationship between soil nutrients and soil microbial biomass nitrogen

**Figure 9:**
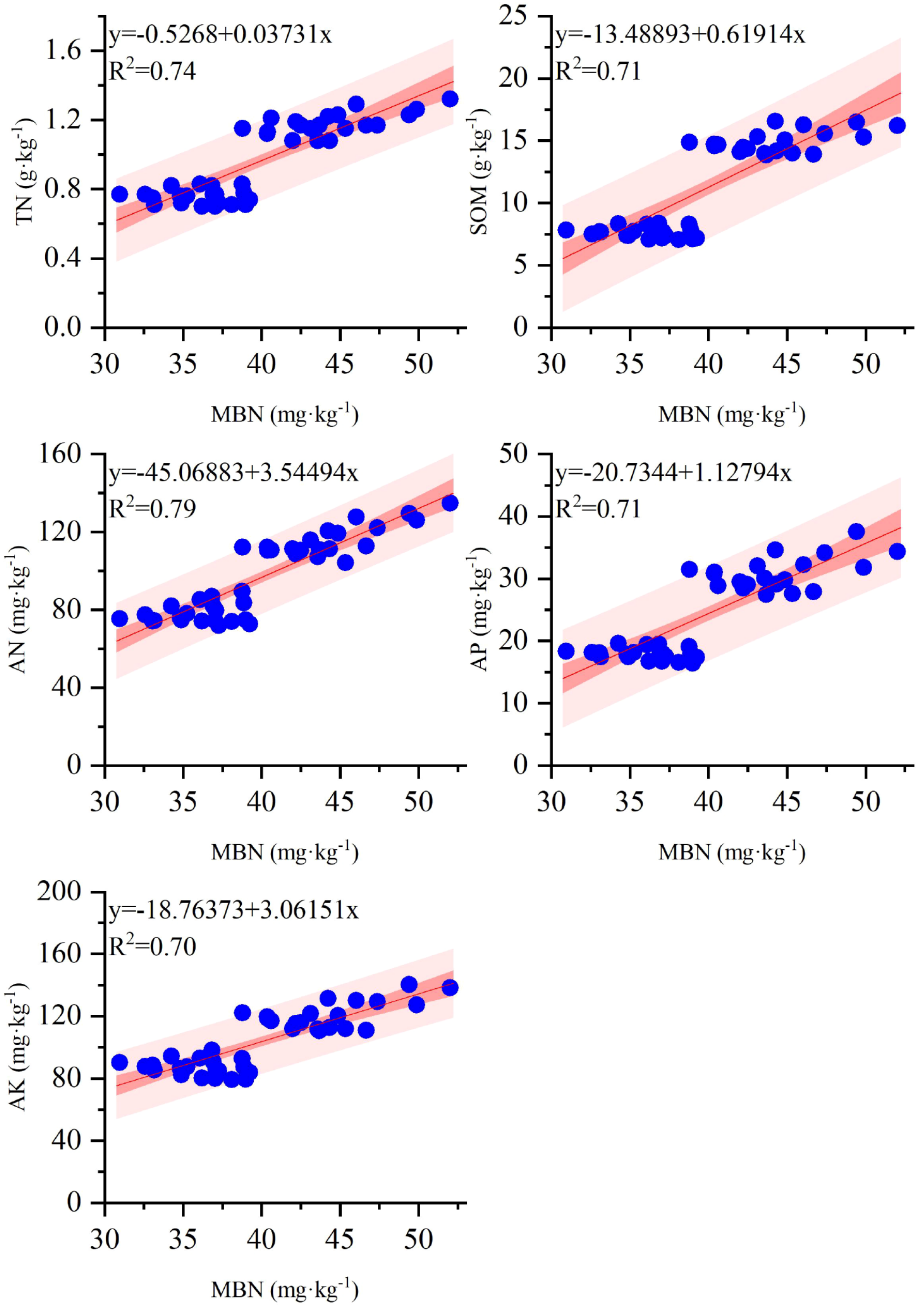
The correlation between soil total nitrogen, organic matter, alkali-hydrolyzed nitrogen, available phosphorus, available potassium and soil microbial biomass nitrogen in 0−40 cm soil depth. TN, SOM, AN, AP, AK, and MBN represent soil total nitrogen, soil organic matter, alkali-hydrolyzed nitrogen, available phosphorus, available potassium and soil microbial biomass nitrogen, respectively.

In 2021-2023, the relationship between MBN and TN, SOM, AN, AP, and AK under ridge-furrow planting patterns with different ridge-furrow ratios was systematically studied (**Figure 9**). Correlation analysis revealed a significant positive correlation between MBN and TN, SOM, AN, AP and AK. It shows that increasing soil nutrient content is conducive to promoting the increase of soil microbial biomass nitrogen content.

### 3.4 Grain filling characteristics

**Figure 10:**
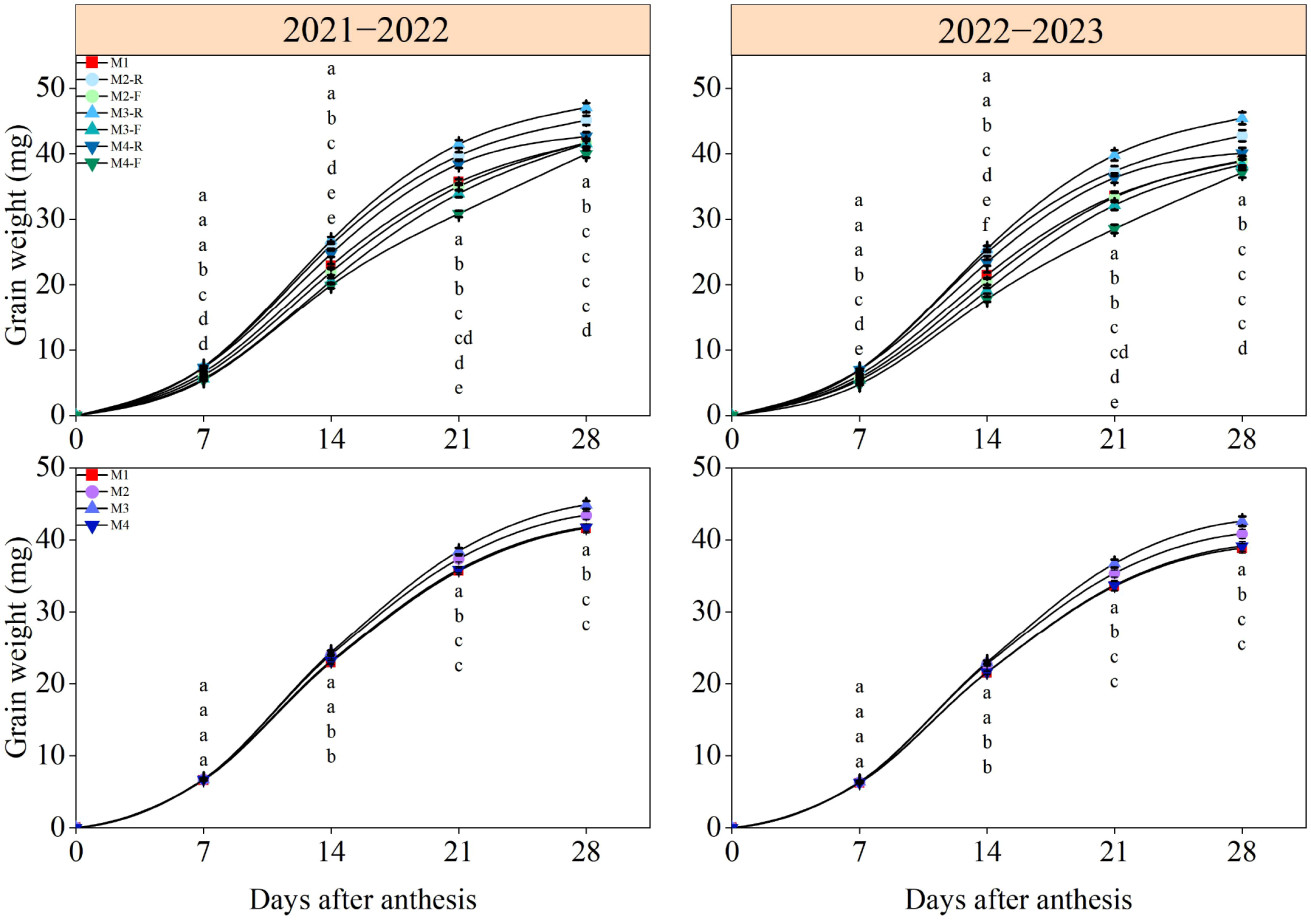
Effects of different treatments on grain weight of wheat in 2021−2022 and 2022−2023. -R and -F denote the ridge and furrow of the treatment, respectively. Distinct letters following the data values represent statistically significant differences across treatments at *P* < 0.05 (n = 3). Vertical bars denote the mean ± standard error.

**Figure 11:**
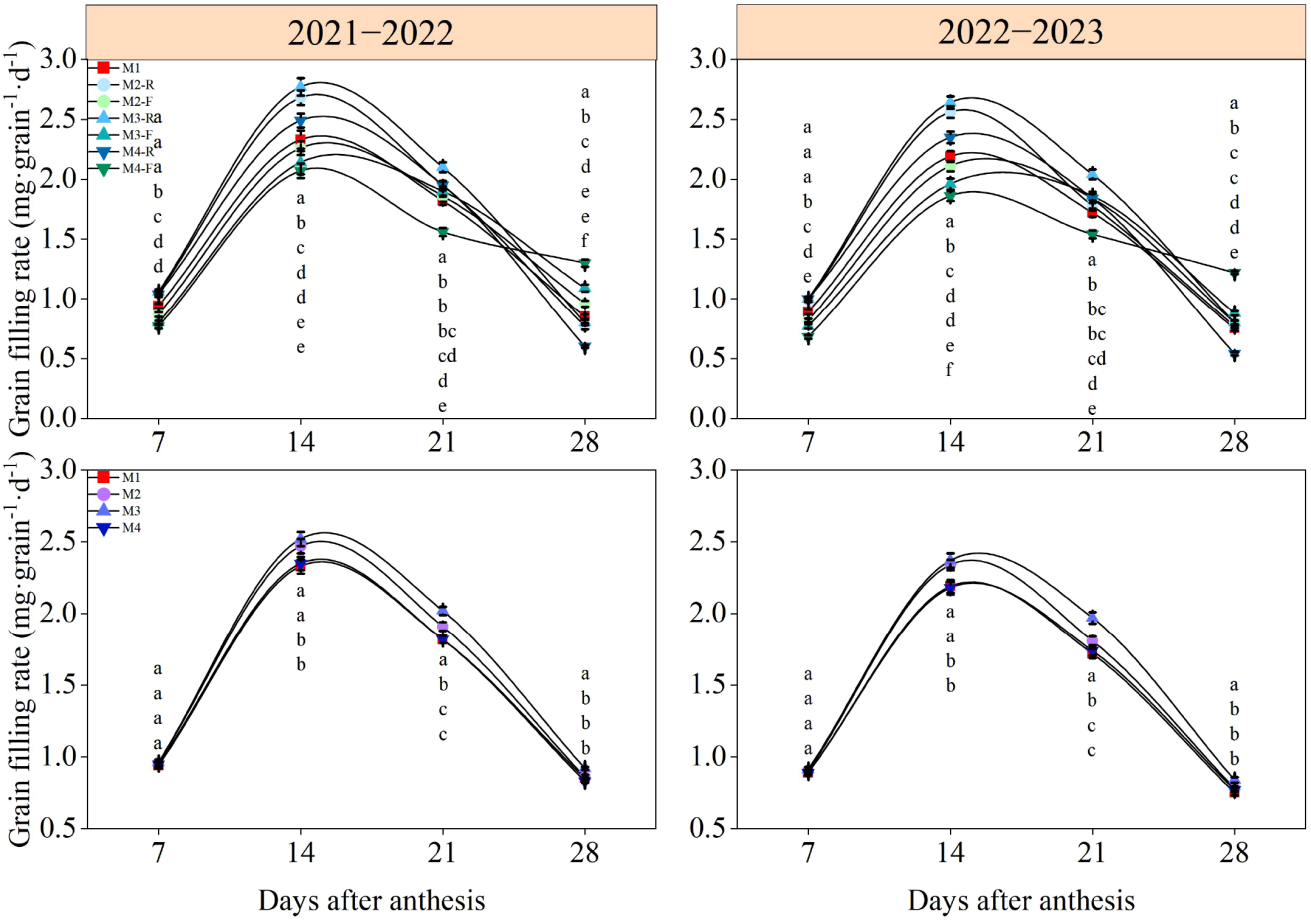
Effects of different treatments on grain filling rate of wheat in 2021−2022 and 2022−2023. -R and -F denote the ridge and furrow of the treatment, respectively. Distinct letters following the data values represent statistically significant differences across treatments at *P* < 0.05 (n = 3). Vertical bars denote the mean ± standard error.

Wheat grain weight exhibited a continuous increasing trend after anthesis, whereas the grain filling rate showed a pattern of increasing first followed by decreasing (**Figures 10**, **11**). At 7 days post-anthesis, grain weight and filling rate were significantly elevated in the M2-R, M3-R, and M4-R treatments relative to the M1, M2-F, M3-F, and M4-F treatments. No significant differences in grain weight and filling rate were found across M1–M4 treatments at this stage. Fourteen days following anthesis, grain weight and filling rate under M2-R and M3-R surpassed those of all other treatments by a statistically significant margin (*P* < 0.05). Meanwhile, the corresponding indices under the M2 and M3 treatments were significantly greater than those under the M1 and M4 treatments (*P* < 0.05). At 21 and 28 days after anthesis, grain weight values in the M3-R treatment were significantly elevated relative to those in all other tested treatments (*P* < 0.05). With respect to grain filling rate, values under the M3-R treatment were significantly greater than those of other treatments at 21 days post-anthesis, while the M3-F treatment exhibited the highest filling rate among all treatments at 28 days post-anthesis (*P* < 0.05). In the 2021−2022 growing season, the grain filling rate in the M3 exceeded that of other treatments by 5.92–10.76% and 6.18–9.75% at 21 days and 28 days post-anthesis, respectively. In the 2022−2023 growing season, the respective increments in grain filling rate under the M3 treatment relative to other treatments were 8.73−14.28% and 6.83−11.19% at the two aforementioned stages.

Fitting equations and their parameters per treatment are given in **Table 2**. The M4-F treatment had the highest values of Tmax and T; however, no statistically significant discrepancy was detected between the M4-F and M3-F in the 2021−2022. Across both growing seasons, the Vmax and Vmean values under the M3-R treatment were significantly higher than those in all other treatments (*P* < 0.05). In the 2021−2022 growing season, Vmax values in the M3 exceeded those in the other three treatments by 7.84%, 3.59%, and 7.58%, respectively, while the Vmean was 6.82%, 3.22%, and 6.56% higher than the corresponding values of these three treatments. In the 2022−2023, the Vmax under the M3 exceeded that of the M1, M2, and M4 treatments by 9.93%, 4.77%, and 9.04%, respectively, and the Vmean was 8.88%, 4.45%, and 7.82% higher than that of the M1, M2, and M4 treatments, respectively. This shows that M3 treatment can effectively enhance the wheat grain filling rate and thereby increase the final grain weight.

**Table 2:** Effects of various treatments on grain filling models and key filling parameters of wheat.

Year	Treatment		Growth curve equation		Vmax (mg·grain^-1^·day^-1^)		Tmax (day)		Vmean (mg·grain^-1^·day^-1^)		T (day)	
2021−2022	M1	–	y=41.96/(1+32.27e^-0.26x^)	y=41.96/(1+32.27e^-0.26x^)	2.73c	2.73c	13.36c	13.36a	1.35c	1.35c	31.04d	31.04a
	M2	R	y=45.19/(1+35.16e^-0.27x^)	y=43.68/(1+33.85e^-0.26x^)	3.05b	2.84b	13.18cd	13.55a	1.50b	1.40b	30.20d	31.22a
		F	y=42.28/(1+32.83e^-0.25x^)		2.64cd		13.97b		1.31d		32.35c	
	M3	R	y=47.24/(1+36.63e^-0.27x^)	y=45.25/(1+34.85e^-0.26x^)	3.19a	2.94a	13.34c	13.66a	1.56a	1.44a	30.36d	31.33a
		F	y=42.64/(1+32.78e^-0.24x^)		2.56d		14.54a		1.27e		33.69b	
	M4	R	y=43.01/(1+35.19e^-0.28x^)	y=41.89/(1+36.13e^-0.26x^)	3.01b	2.73c	12.72d	13.36a	1.48b	1.36c	29.13e	31.03a
		F	y=41.44/(1+26.50e^-0.22x^)		2.28e		14.90a		1.16f		35.78a	
2022−2023	M1	–	y=39.16/(1+32.91e^-0.26x^)	y=39.16/(1+32.91e^-0.26x^)	2.55cd	2.55c	13.44cd	13.44a	1.26c	1.26c	31.11cd	31.11a
	M2	R	y=42.91/(1+34.58e^-0.27x^)	y=41.09/(1+34.71e^-0.26x^)	2.88b	2.67b	13.12d	13.64a	1.42b	1.31b	30.14de	31.32a
		F	y=39.53/(1+35.58e^-0.26x^)		2.57c		13.74c		1.26c		31.41c	
	M3	R	y=45.43/(1+36.76e^-0.27x^)	y=43.05/(1+35.59e^-0.26x^)	3.08a	2.80a	13.35d	13.74a	1.50a	1.37a	30.37cd	31.41a
		F	y=39.28/(1+34.69e^-0.25x^)		2.46d		14.19b		1.21d		32.57b	
	M4	R	y=40.48/(1+34.93e^-0.28x^)	y=39.48/(1+32.47e^-0.26x^)	2.83b	2.57c	12.69e	13.39a	1.39b	1.27c	29.10e	31.06a
		F	y=38.85/(1+28.08e^-0.22x^)		2.14e		15.16a		1.08e		36.05a	

R and F represent the ridge and furrow of the treatment, respectively. The significant difference between treatments with *P* < 0.05 was indicated by the addition of different letters after the value (n=3).

### 3.5 Grain starch accumulation

**Figure 12:**
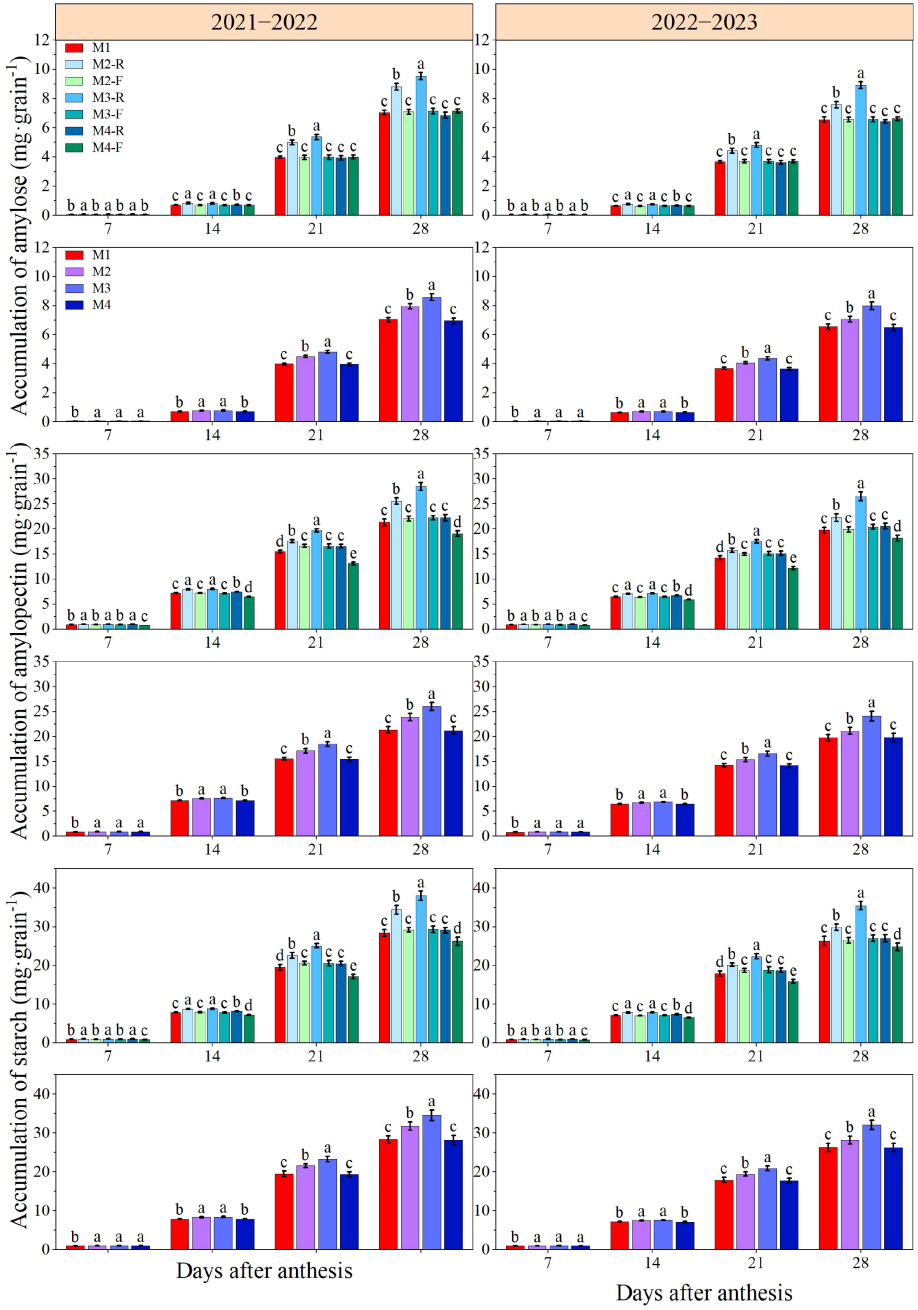
Effects of different treatments on grain starch content of wheat in 2021−2022 and 2022−2023. -R and -F denote the ridge and furrow of the treatment, respectively. Distinct letters following the data values represent statistically significant differences across treatments at *P* < 0.05 (n = 3). Vertical bars denote the mean ± standard error.

Across both growing seasons, the starch accumulation in wheat grains showed a continuous increasing trend after anthesis (**Figure 12**). At 7 and 14 days after anthesis, ridge planting significantly increased grain amylose, amylopectin, and total starch accumulation compared with the M1 and furrow planting (*P* < 0.05). During both growing seasons, M3-R significantly promoted the accumulation of grain amylose, amylopectin, and total starch compared with all other treatments at 21 and 28 days post-anthesis (*P* < 0.05). In the 2021−2022 growing season, compared with the M1, M3 induced increases of 6.08%, 5.33%, and 5.38% in grain amylose, amylopectin, and total starch accumulations at 7 days after anthesis, respectively, with the corresponding increments reaching 10.10%, 6.96%, and 18.86% at 14 days after anthesis. In the 2022−2023, M3 elevated the accumulations of amylose, amylopectin, and total starch by 6.77%, 5.49%, and 5.56% at 7 days after anthesis, and by 10.31%, 5.44%, and 5.88% at 14 days after anthesis, versus the M1. No significant differences were detected in amylose, amylopectin, or total starch accumulations between the M3 and the M2 and M4 at 7 and 14 days after anthesis; additionally, there was no significant difference between the M3 and M2 treatments at 14 days after anthesis. In the 2021−2022, the grain amylose accumulation under the M3 was 7.21−21.94% and 7.87−23.37% higher than that under other treatments at 21 and 28 days after anthesis, respectively. The corresponding increments in the 2022−2023 were 7.15−19.36% and 12.93−23.06%, respectively. Moreover, M3 consistently maintained significantly higher grain amylopectin accumulation than all other treatments at 21 and 28 days post-anthesis across both growing seasons (*P* < 0.05). With respect to total starch accumulation, the M3 led to increases of 7.72−20.18% and 8.74−22.88% compared with other treatments at 21 and 28 days after anthesis in the 2021−2022, respectively. In the 2022−2023, the total starch accumulation under the M3 treatment was 7.66−17.64% and 13.84−22.07% higher than that under other treatments at the two aforementioned stages, respectively. This indicated that M3 treatment effectively promoted the synthesis of amylose and amylopectin, thereby increasing the starch content of wheat grains.

### 3.6 Grain starch related enzyme activity

**Figure 13:**
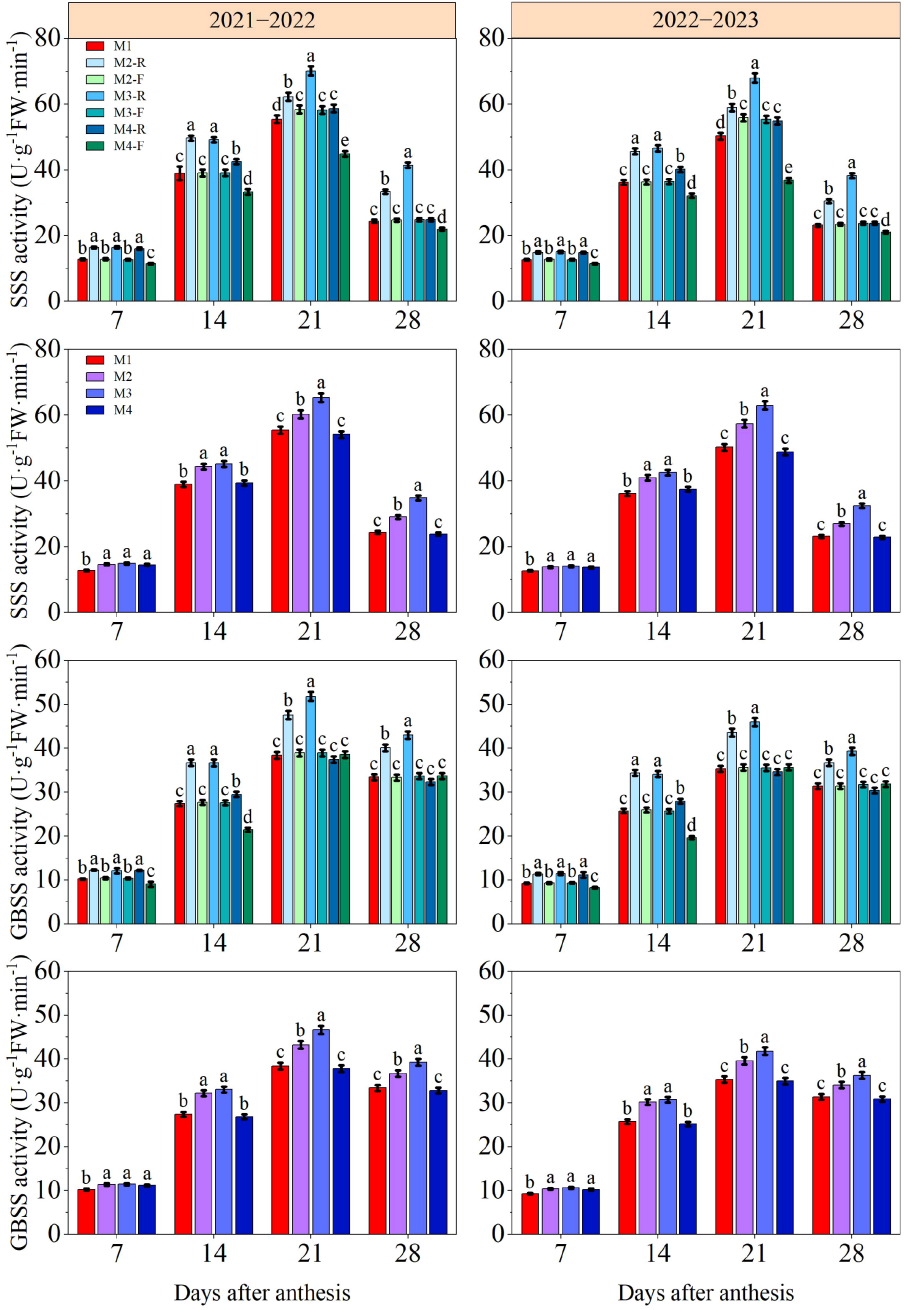
The effects of different treatments on SSS activity and GBSS activity of wheat grains in 2021−2022 and 2022−2023. -R and -F denote the ridge and furrow of the treatment, respectively. Distinct letters following the data values represent statistically significant differences across treatments at *P* < 0.05 (n = 3). Vertical bars denote the mean ± standard error.

Across both growing seasons, the activities of SSS and GBSS in wheat grains exhibited a trend of increasing first and then decreasing after anthesis (**Figure 13**). At 7 and 14 days after anthesis, ridge treatments exhibited markedly higher grain SSS and GBSS activities compared with the M1 and furrow treatments (*P* < 0.05). During both growing seasons, the SSS and GBSS activities in grains under M3-R were significantly superior to those under all other treatments at 21 and 28 days after anthesis (*P* < 0.05). At 7 days after anthesis across the two growing seasons, the SSS and GBSS activities under all ridge-furrow planting treatments were significantly higher than the M1, whereas no significant differences were detected among the various ridge-furrow planting treatments themselves. At 14 days after anthesis, both SSS and GBSS activities under M3 were markedly higher than the corresponding values observed under M1 and M4 (*P* < 0.05), while no significant variation was found between M3 and M2 treatments. In the 2021−2022, the SSS and GBSS activities under the M3 treatment were 8.36−45.81% and 7.86−23.42% higher than those under other treatments, respectively, at 21−28 days after anthesis. The corresponding increments in the 2022−2023 growing season were 9.65−42.06% and 5.51−19.51%, respectively. This shows that M3 treatment exerts a positive effect on starch synthesis in grains by upregulating the activity of associated enzymes.

### 3.7 Grain yield and its components

Varied ridge-furrow ratios in this planting configuration significantly affected wheat yield components (*P* < 0.05) (**Table 3**). Across both growing seasons, spike number and 1000-grain weight for M3 treatment exceeded those of all other treatments by a significant margin (*P* < 0.05). By contrast, grain number per spike showed no significant variation across all treatments over the two growing seasons. In the 2021−2022, compared with the M1, the grain yields under the M2 and M3 treatments were increased by 4.44% and 7.70%, respectively. The corresponding yield increments in the 2022−2023 growing season reached 4.56% and 9.57%, respectively. Nevertheless, there were no statistically significant differences in grain yield between M1 and M4 treatments throughout the two growing seasons. Collectively, these results indicate that the M3 achieves an increase in wheat grain yield through the synergistic improvement of spike number and grain weight.

**Table 3:** Differences of wheat grain yield and its components under different treatments.

Year	Treatment		Spike Number (10^4^·hm^-2^)		Kernel Number per Spike		Thousand Kernel Weight (g)		Yield (kg·hm^-2^)	
2021−2022	M1	–	657.9c	657.9b	36.91bc	36.91a	44.38d	44.38c	9101b	9101c
	M2	R	654.6c	658.6b	37.30ab	37.35a	47.46b	45.68b	9813a	9505b
		F	662.6c		37.40ab		43.89d		9197b	
	M3	R	663.5c	675.2a	36.23c	37.19a	48.50a	46.51a	9851a	9802a
		F	692.7b		38.23a		43.53d		9730a	
	M4	R	605.2d	642.6c	36.70bc	36.50a	45.44c	44.40c	8644c	9019c
		F	717.5a		38.11a		42.33e		9769a	
2022−2023	M1	–	612.1bc	612.1b	40.20a	40.20a	40.45d	40.45c	8538c	8538c
	M2	R	604.9c	614.9b	40.64a	40.23a	44.05b	42.10b	9270b	8927b
		F	624.9b		39.81a		40.15d		8584c	
	M3	R	613.4bc	631.5a	40.24a	39.90a	46.12a	43.59a	9707a	9355a
		F	658.7a		39.40a		39.80d		8827b	
	M4	R	548.4d	588.4c	39.84a	39.67a	43.12c	42.05b	8148d	8435c
		F	668.4a		39.32a		39.90d		9009b	

R and F represent the ridge and furrow of the treatment, respectively. The significant difference between treatments with *P* < 0.05 was indicated by the addition of different letters after the value (n=3).

### 3.8 Correlation analysis

**Figure 14:**
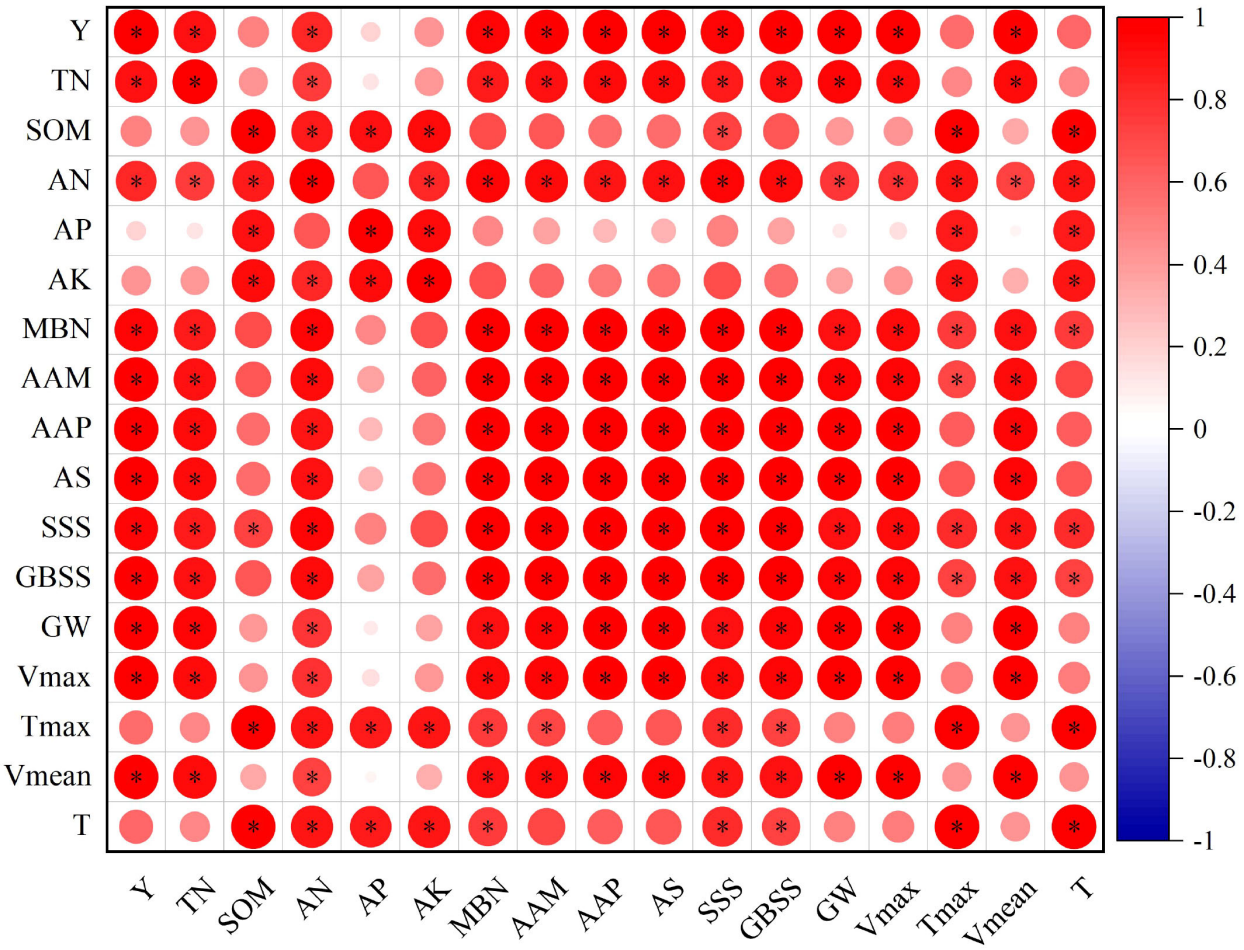
Correlation of grain yield, soil total nitrogen, soil organic matter, soil available nitrogen, soil available phosphorus, soil available potassium, soil microbial biomass nitrogen content, grain amylose content, grain amylopectin content, grain total starch content, grain soluble amylase activity, grain bound starch synthase, grain weight, maximum grain filling rate, time to maximum grain filling rate, average grain filling rate and grain filling time under different treatment conditions. * represents a significant correlation at the *P* < 0.05 level. Y, TN, SOM, AN, AP, AK, MBN, AAM, AAP, AS, SSS, GBSS, GW, Vmax, Tmax, Vmean and T represented grain yield, soil total nitrogen, soil organic matter, soil available nitrogen, soil available phosphorus, soil available potassium, soil microbial biomass nitrogen, average amylose accumulation after anthesis, average amylopectin accumulation after anthesis, average starch accumulation after anthesis, average soluble amylase activity after anthesis, average bound starch synthase after anthesis, average grain weight after anthesis, maximum grain filling rate, time to maximum grain filling rate, average grain filling rate and grain filling time, respectively.

The correlation coefficient was used to analyze the relationship between wheat traits (**Figure 14**). Y was positively correlated with TN, AN, MBN, AAM, AAP, GS, SSS, GBSS, GW, Vmax and Vmean (*P* < 0.05). AAM, AAP and GS were significantly positively correlated with TN, AN, MBN, SSS, GBSS, GW, Vmax and Vmean (*P* < 0.05), and AAM was also significantly positively correlated with Tmax and T (*P* < 0.05). The results showed that the increase of grain yield could be achieved by increasing the content of total nitrogen, alkali-hydrolyzable nitrogen and microbial biomass nitrogen in soil, increasing grain starch synthesis and promoting grain filling.

**Figure 15:**
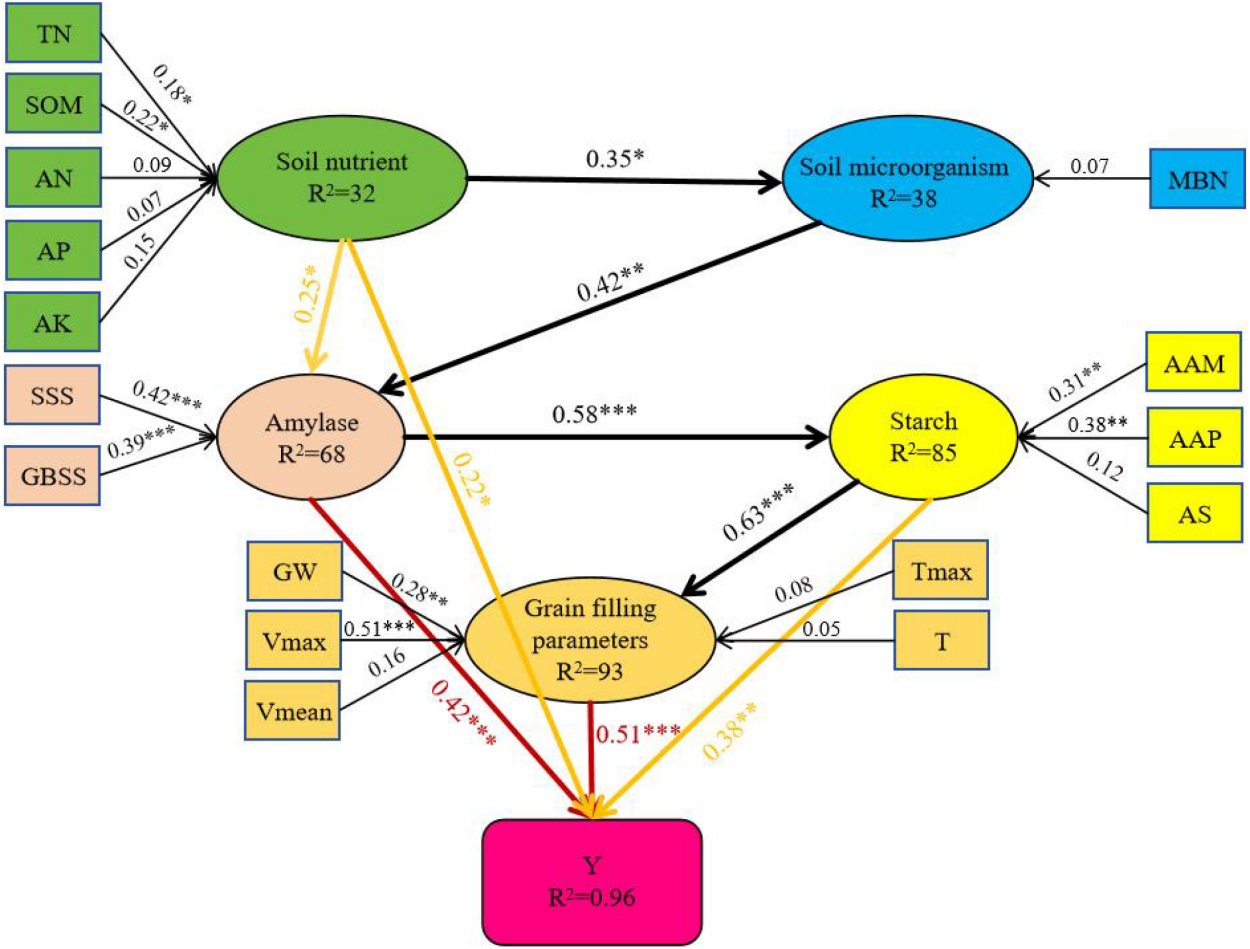
SEM path diagram of soil nutrients, microorganisms, starch synthesis and filling parameters on yield. The ellipse is the latent variable, and the rectangle is the explicit variable. The path side value is the path coefficient, and R2 is the adjusted determination coefficient. *p < 0.05, **p < 0.01, ***p < 0.001. Y, TN, SOM, AN, AP, AK, MBN, AAM, AAP, AS, SSS, GBSS, GW, Vmax, Tmax, Vmean and T represented grain yield, soil total nitrogen, soil organic matter, soil available nitrogen, soil available phosphorus, soil available potassium, soil microbial biomass nitrogen, average amylose content after anthesis, average amylopectin content after anthesis, average total starch content after anthesis, average soluble amylase activity after anthesis, average bound starch synthase after anthesis, average grain weight after anthesis, maximum grain filling rate, time to maximum grain filling rate, average grain filling rate and grain filling time, respectively.

SEM path analysis explained the different factors affecting the yield (**Figure 15**). The positive driving effect of soil nutrients on microorganisms and amylase was significant. Soil nutrients regulated amylase activity, and amylase had a strong promoting effect on starch accumulation (0.58, P < 0.001). Starch accumulation further significantly regulated grain filling parameters (0.63, P < 0.001). At the same time, the direct contribution of filling parameters to yield was the strongest (0.51***), followed by amylase (0.42***), starch (0.38**) and soil nutrients (0.22 *). These findings highlight the need to regulate soil nutrient content to promote wheat starch synthesis, increase grain filling rate, and increase yield.

## 4 Discussion

### 4.1 Responses of soil microbial biomass nitrogen and nutrient contents to ridge-furrow ratios

Soil microorganisms are directly engaged in critical ecological processes and exhibit significant correlations with nutrient recycling, organic matter breakdown, soil aggregate formation, and soil fertility. Consequently, they are widely regarded as the most active component of soil systems (Pang et al., 2019). Currently, soil microbial biomass nitrogen content is recognized as a key indicator for assessing soil microbial status (Santos et al., 2023). Previous research has demonstrated that the ridge-furrow planting pattern indirectly dominates the migration path, accumulation efficiency and effectiveness of soil nutrients by changing the water movement process of farmland, and affects soil microbial biomass (Xu et al., 2025). The present study found that, in comparison to other treatments, the M3 treatment not only significantly increased soil MBN content but also elevated the concentrations of TN, SOM, AN, AP, and AK in the 0−20 cm soil depth. Notably, no significant difference in organic matter content within the 0−20 cm soil depth was observed between the M3 and M2 treatments, which aligns with findings from previous studies (Yang et al., 2024). These results indicate that the M3 treatment can effectively enhance soil nutrient content and sustain nutrient cycling. It is mainly because the ridge-furrow ratio affects the micro-topographic characteristics of the soil, which in turn affects the rainwater infiltration and the distribution of water fields (Li et al., 2019). Suitable ridge-furrow planting patterns can improve the soil microenvironment and optimize water movement (Wang et al., 2017). Optimized water movement reduce nutrient loss and enhance nutrient availability. These changes create a suitable living environment for microorganisms, increase soil nutrient content and effectiveness, and stimulate soil microbial activities (Lai et al., 2024; Gu et al., 2021). The increase of soil microbial activity may increase the decomposition of straw. Soil microorganisms decompose complex organic components in straw (e.g., cellulose, hemicellulose, and lignin) into simple compounds. This process relies on the synthesis and secretion of enzymes and other bioactive substances. This decomposition process not only accelerates nutrient release but also increases soil organic matter content, thereby significantly improving soil fertility (Ma et al., 2025). Consistent with this mechanism, the correlation analysis in the present study (**Figure 9**) between soil MBN content and the concentrations of TN, SOM, AN, AP, and AK further supported this perspective. In conclusion, M3 treatment can effectively increase soil nutrient content, regulate soil microbial community structure, and ensure sufficient nutrient supply in the later growth stage of wheat, thus promoting the filling process of wheat. However, this experiment only collected soil samples in the 0−40 cm soil depth in the North China Plain, reflecting the main characteristics of the soil in the study area, but could not fully characterize the deep soil properties. The follow-up study can add deep soil sampling gradient, take into account the characteristics of surface and deep soil, fully reflect the overall situation of soil, and provide a more scientific basis for the sustainable management of farmland.

### 4.2 Responses of grain filling characteristics of wheat to ridge-furrow ratios

Grain filling is a key agronomic trait of winter wheat, and optimizing its quality is crucial for improving grain weight and yield (Wu et al., 2025). As a complex physiological process, grain filling is influenced by soil moisture and nutrient management (Liu et al., 2024). Previous studies have demonstrated that optimized tillage practices can improve the soil hydrothermal environment, reduce nutrient loss, enhance soil physical and chemical properties, and thereby increase crop yields in farmland ecosystems (Wang et al., 2025a). Therefore, adjusting tillage methods to improve grain filling parameters may serve as an effective approach to boost grain yield. The results of this experiment showed that grain weight and grain filling rate first increased and then decreased with increasing ridge-furrow ratio. Compared with the other treatments, M3 significantly elevated Vmax and Vmean, thereby contributing to a marked improvement in grain weight. This indicates that an appropriate ridge-furrow ratio accelerate photosynthate accumulation and maintains a high grain filling rate after anthesis. The primary mechanism is that the ridge-furrow planting pattern modifies the field microtopography and soil properties in both ridges and furrows, thereby optimizing soil moisture and nutrient fluxes (Han et al., 2025). Specifically, the ridge-furrow ratio significantly affects the distribution and redistribution of soil water and crop growth. An appropriate ridge-furrow configuration maintains soil moisture stability through rainwater harvesting and water retention during the filling stage, ensuring the coordinated development of photosynthetic source strength and seed sink capacity (Wang et al., 2025c). Meanwhile, sufficient soil moisture promotes the dissolution and mobility of various nutrients, effectively increasing soil nutrient availability and providing adequate water and nutrients for wheat grain filling (Borut et al., 2010). The M3 treatment well coordinated the water distribution between ridges and furrows, increased sufficient water for the grain filling of wheat on ridges, and increased the grain filling rate (**Table 2**). In contrast, the M4 treatment may lead to uneven water distribution due to excessive ridge width, resulting in a dry center in the ridges and localized water stress. Under water stress conditions, the effective grain filling period of ridge-grown wheat is shortened, ultimately reducing the grain filling rate (Chen et al., 2011; Shi et al., 2021).

Starch and nutrient accumulation in the endosperm during grain filling directly governs crop yield and grain quality. Starch is the main storage compound in wheat reproductive organs, accounting for approximately 65%−70% of the total grain weight (KyungHee and JaeYoon, 2021). In this study, compared with other treatments, the M3 treatment significantly increased amylose accumulation, amylopectin accumulation, and total starch accumulation by enhancing the activities of SSS and GBSS. This is consistent with the findings of Liang et al. (2021), who reported that SSS and GBSS activities in wheat grains grown on ridges are enhanced under mild drought stress. However, severe water deficit significantly reduces the accumulation of amylose, amylopectin, and total starch due to limited photosynthate supply (Zib et al., 2025). Additionally, our previous research (Liu et al., 2023) has shown that the vertical height difference between adjacent ridges and furrows alters the individual niche of plants and affects the competition for light resources. An appropriate ridge-furrow ratio promotes crop photosynthesis, thereby facilitating starch accumulation.

### 4.3 Responses of wheat grain yield and its components to ridge-furrow ratios

Increasing grain yield remains the primary goal of wheat production. Favorable growth conditions can promote the healthy growth of crops, thereby improving yield per unit area (Dong et al., 2025). Studies have indicated that appropriate tillage methods help improve soil structure and fertility, and further promote crop growth and yield (Li et al., 2025). A large body of research has demonstrated that ridge-furrow planting patterns can increase crop yields (Lai et al., 2024; Gu et al., 2021; MakMensah et al., 2021). The results of this study indicated that grain yield under the M3 treatment was 7.70–9.57% higher than that under the M1 treatment, with M3 achieving the maximum grain yield across all treatments. This is attributed to its alternating ridge-furrow structure, which improves soil physical and chemical properties and increases nutrient content, thereby enhancing crop yield (Cui et al., 2025). However, this experiment found that the grain yield of the M4 treatment did not increase compared with the M1 treatment. This is because wheat grown on ridges experiences higher temperature stress during the filling stage; insufficient distribution of nutrients and water on ridges causes premature leaf senescence, which reduces photosynthesis and the accumulation of photosynthetic products, ultimately leading to decreased crop yield (Pant et al., 2025).

Higher yields rely on the coordinated optimization of crop yield components. Studies have demonstrated that ridge-furrow planting increases panicle number per unit area, grain number per panicle and grain weight relative to flat planting (Liang et al., 2024). In our study, the higher yield achieved by the M3 treatment compared with M1 was mainly due to the simultaneous increase in spike number and grain weight. This is primarily attributed to the fact that the ridge-furrow planting pattern can significantly improve water conditions and effectively increase the number of spikes (Fang et al., 2022). Meanwhile, the ridge-furrow planting pattern provides sufficient water and nutrients during grain filling, delays leaf senescence, and thus increases grain weight (Ali et al., 2018). In addition, the ridge-furrow planting pattern results in the zonal distribution of wheat in the field; wheat near the furrows exhibits marginal advantages, which play a positive role in regulating yield components (Xu et al., 2022).

## 5 Conclusions

This study was conducted to investigate the impacts of ridge-furrow planting patterns with different ridge-furrow ratios on grain filling characteristics, soil nutrient content, and grain yield of winter wheat in the NCP. The results showed that, compared with other treatments, M3 treatment (75∶50 cm ridge-furrow planting pattern) increased soil nutrients, promoted microbial accumulation, provided sufficient nutrient supply for grain filling, and stimulated amylase activity. At the same time, M3 treatment increased the accumulation of amylose and amylopectin by increasing SSS and GBSS activities at the filling, promoted wheat grain filling, increased grain weight and Vmax, and finally obtained the highest grain yield. Therefore, the findings of this study provide a theoretical foundation and practical reference for determining the appropriate ridge-furrow ratio in ridge-furrow planting patterns suitable for winter wheat production in the NCP. However, for regions other than NCP, the design of a reasonable ridge-furrow ratio for ridge-furrow planting patterns should take into account soil types and rainfall conditions, which requires further research. In the next step, research will focus on the integration of sowing machinery and agronomic practices based on the optimal ridge-furrow ratio identified in this study.

## Data Availability

The raw data supporting the conclusions of this article will be made available by the authors, without undue reservation.
